# Patient Dimensions From a Single CT Image Provide a Clinically Practical and Accurate Method for Dose Estimation in Canine Abdominal CT Imaging

**DOI:** 10.1111/vru.70223

**Published:** 2026-07-29

**Authors:** Claire Whittaker, Monique Mayer, Matthew Hutcheson, Sally Sukut

**Affiliations:** ^1^ Small Animal Clinical Sciences Western College of Veterinary Medicine University of Saskatchewan Saskatoon Saskatchewan Canada

**Keywords:** conversion factors, dose assessment methods, veterinary radiology

## Abstract

Size‐specific dose estimate (SSDE) uses linear dimensions measured on a computed tomographic (CT) image to scale the scanner‐reported volumetric computed tomography dose index (CTDI_vol_) and thereby estimate dose. The objectives of this retrospective observational study were to evaluate whether the American Association of Physicists in Medicine (AAPM) geometric size metrics for abdominal CT, when implemented using canine‐specific size measurements, correlate with water‐equivalent diameter (*D*_w_) in canine patients and to compare institutional SSDEs with human benchmarks. Abdominal CT scans from 30 dogs across three body weight categories (<10, 10–30, and >30 kg) were analyzed. The relationships between effective diameters calculated using four geometric metrics—dorsoventral dimension (DV), lateral dimension (LAT), the sum of DV and LAT, and the square root of their product—and *D*_w_, which incorporates both size and attenuation, were analyzed using linear regression. The strongest correlation with *D*_w_ was observed for the combined DV and LAT metrics (*R* ^2^ 0.94, *p* < 0.001). The displayed CTDI_vol_ underestimated SSDE by 50%. The 75th percentile of the SSDE for a single‐phase, pre‐contrast abdominal examination was 21 mGy, two to four times higher than the US human dose reference level. In conclusion, geometric size metrics measured from a single CT image were in close agreement with attenuation‐based SSDE (*D*_w_) for canine abdominal CT scans, supporting the feasibility of applying the AAPM SSDE estimation method to canine abdominal CT imaging. These findings demonstrate the importance of incorporating patient size into CT dose estimation and suggest that institutional SSDE values may warrant dose optimization in veterinary abdominal CT practice.

**Abbreviations**
AD: achievable dose
CTDI_vol_
: volumetric computed tomography dose index
DRL: diagnostic reference level
DV: dorsoventral dimension
*D*_w_
: water‐equivalent diameter
LAT: lateral dimension
SSDE: size‐specific dose estimate

## 1 Introduction

Computed tomography (CT) is a diagnostic imaging modality that delivers relatively high doses of ionizing radiation and is increasingly utilized in veterinary medicine for the diagnosis, treatment planning, and monitoring of a wide range of diseases [1]. The increasing use of CT raises concern about veterinary patient radiation exposure and the potential risk of radiation‐induced malignancies [2]. It has been hypothesized that the accelerated aging of dogs relative to the human species may allow for the development of radiation‐induced cancers within their natural lifespan [2]. This hypothesis is supported by studies that found that the time to cancer induction is inversely proportional to cell division rates in exposed tissues, which, in turn, are inversely related to species lifespan [3, 4, 5].

To optimize patient radiation dose, CT scanner manufacturers have been required since 2002 to display the volumetric computed tomography dose index (CTDI_vol_), a standardized metric to compare radiation output between different scanners [6]. CTDI_vol_ is a single dose parameter representing the average dose within the scan volume for a standardized phantom [7]. For abdominal imaging using body scan protocols or body bow‐tie filters, CT scanner vendors use a 32 cm diameter phantom [8]. A limitation of CTDI_vol_ is that it considers only the scanning parameters (tube voltage, tube current, gantry rotation time, pitch, and bow‐tie filter selection), not patient size, and, therefore, does not estimate patient dose [9, 10]. Interpretation of CTDI_vol_ as dose could lead to underestimation of smaller patient dose levels by a factor of 2–3 if the 32 cm PMMA phantom is used for reference [8].

To address the limitations of CTDI_vol_, the American Association of Physicists in Medicine (AAPM) introduced the size‐specific dose estimate (SSDE) in 2011 [8]. SSDE provides an estimate of patient dose using linear dimensions measured on the patient or patient image to scale the CTDI_vol_ [8]. Factors to convert CTDI_vol_ to SSDE using anteroposterior, lateral (LAT), and combined patient dimensions were developed to allow routine reporting of patient CT dose estimates with minimal radiologist intervention. However, these patient geometry‐based metrics do not account for x‐ray attenuation, which is more relevant than patient size in determining absorbed dose. To provide a more accurate alternative, AAPM Report 220 developed the water‐equivalent diameter (*D*_w_), a metric measured from CT images that considers both patient size and x‐ray attenuation, and compared it with the geometric‐based metrics [11]. The report concluded that x‐ray attenuation had a lower impact in the abdomen compared to the thorax, and that using patient geometric dimensions instead of an attenuation‐based metric to calculate SSDE in abdominal CT imaging was a reasonable alternative. The AAPM conversion factors were developed using human data, and their applicability to canine patients with different body conformations has not been previously evaluated. Given the differences in body size and shape between human and canine patients, this study aimed to evaluate the feasibility of applying the AAPM geometric size metrics for abdominal CT to canine patients using canine‐specific size measurements.

The primary objective of this retrospective, observational study was to determine which geometric size metric best estimates *D*_w_‐based SSDE in canine abdominal CT. A secondary objective was to evaluate patient radiation dose using a weight‐based abdominal CT protocol that adjusted x‐ray tube current and scanning rotation time (mAs) to mitigate excessive CT dose to smaller sized dogs.

## 2 Materials and Methods

### 2.1 Patient Population

Thirty canine patients were retrospectively identified from the Western College of Veterinary Medicine Veterinary Medical Centre medical records via a reverse chronological search beginning March 1, 2023; all had undergone abdominal CT on an 80‐slice sliding‐gantry CT scanner (Aquilion Prime SP, Canon Medical Systems, Markham, ON). Inclusion criteria included canine species, abdominal scan, acquisition slice thickness ≤5 mm, body weight recorded on the same day the scan was performed, and a recorded scan CTDI_vol_. Exclusion criteria were applied on review of CT images using a picture archiving and communication system (PACS) (McKesson Radiology Station version 12.2, McKesson, San Francisco, CA); scans with a mass in the abdomen and scans in which the field of view did not cover all the patient anatomy were excluded. Patients with an abdominal mass were excluded because the mass could increase the patient diameter measurements used to estimate size, and patients with body regions outside the scan field of view were excluded because information from the entire body contour was used to calculate the water‐equivalent diameter. A medical imaging resident (C.W.) applied the inclusion and exclusion criteria. Patients meeting inclusion and exclusion criteria were consecutively selected to include 10 dogs in each body‐weight category (<10, ≥10–≤30, and >30 kg); these patients were scanned between April 7, 2022, and March 1, 2023.

Patient body weight, breed, age, and sex were recorded from the medical record, and CTDI_vol_ was recorded from the dose report archived with each patient's CT examination in PACS. The CTDI_vol_ is a calculation programmed by the manufacturer to estimate the dose to a standard‐diameter phantom based on the imaging parameters, including kVp, mAs, pitch, and aperture. The CT dosimetry phantom used by the scanner manufacturer was a 32 cm polymethylmethacrylate phantom.

### 2.2 Imaging Parameters

Images were acquired in helical scan mode (pitch factor 0.656, 512 × 512 matrix, tube rotation time 0.5 s, 120 kVp, static mA, slice thickness 1–4 mm) and reconstructed using a soft‐tissue algorithm. At this institution, the mA is varied based on the patient's body weight category to avoid delivering higher radiation doses to smaller patients: 250 (<10 kg), 300 (≥10–≤30 kg), or 400 (>30 kg). The effective mAs (mAs/pitch) values were 381, 457, and 610 for the <10, ≥10–≤30, and >30 kg weight categories, respectively. Patients were scanned in dorsal (*n* = 15) or sternal (*n* = 15) recumbency.

### 2.3 Measurement of Geometric Size Metrics

**FIGURE 1:**
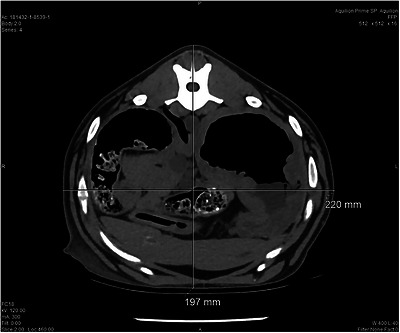
Non‐contrast‐enhanced transverse CT slice of a 25.7 kg dog scanned with an abdominal protocol using 120 kV and 300 mAs. The lateral and dorsoventral dimensions (22.0 and 19.7 cm, respectively, for this patient) were measured on the most cranial transverse slice on which the full transverse process of the first lumbar vertebra was visible.

Four metrics to estimate patient size using cross‐sectional CT image measurements were investigated: (1) dorsoventral (DV) dimension, (2) LAT dimension, (3) the sum of the DV and LAT dimensions, and (4) the square root of the product of the DV and LAT dimensions [8]. The DV and LAT dimensions of the patient were measured once for each patient using digital calipers in an open‐source DICOM viewer (Horos, version 4.0.0RC4; The Horos Project, USA) by a medical imaging resident (C.W.) following review of the measurement technique with a board‐certified radiologist (S.S.). To standardize the *z*‐plane used for size estimation, measurements were obtained on a single axial slice at a consistent anatomic level. Measurements were defined as the maximum diameter of the patient in the DV and right‐to‐left (LAT) directions on the most cranial transverse slice on which the full transverse process of the first lumbar vertebra was visible (Figure 1). Effective diameters were calculated from the DV, LAT, and DV + LAT dimensions for comparison with the water‐equivalent diameter (Supporting Information).

### 2.4 Measurement of Water‐Equivalent Diameter

There is no direct in vivo gold standard for patient absorbed dose from CT [8]. The AAPM considers SSDE based on water‐equivalent diameter (*D*_w_) as the most accurate practical reference, as it incorporates both patient size and x‐ray attenuation [8]. Therefore, in this study, SSDE derived from *D*_w_ was treated as the reference standard against which geometric size‐based SSDE methods were evaluated.

**FIGURE 2:**
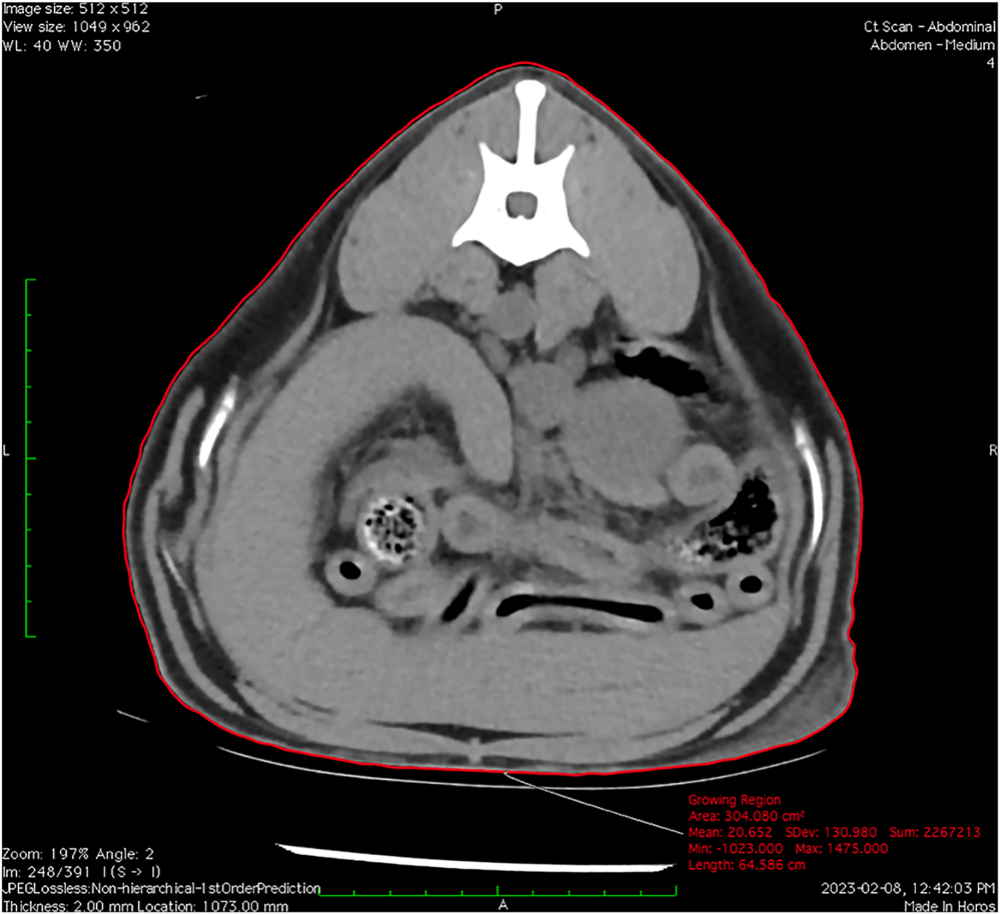
Non‐contrast‐enhanced transverse CT slice of the same dog as in Figure 1. Threshold‐based auto‐contouring using an open‐source medical image viewer was used to generate an ROI for the patient to measure the area (cm^2^) and mean Hounsfield units on each abdominal slice. The water‐equivalent diameter, which considers attenuation in addition to patient size, was then calculated and used to estimate the SSDE for the patient.

A region of interest (ROI) delineating the patient's body was generated using threshold‐based auto‐contouring in an open‐source medical image viewer (Horos, Nimble Co LLC d/b/a Purview, Annapolis, MD) on each transverse, pre‐contrast CT slice from the most cranial image on which the diaphragm was visible to the most cranial image on which the sacroiliac joint was visible (Figure 2). The auto‐contoured ROI was manually edited to conform to patient margins and omit non‐patient sources of attenuation (e.g., CT table) when needed. The ROI area and mean Hounsfield units were recorded. *D*_w_ was calculated for each slice using the following formula recommended in AAPM report 220 [11], where ROI_mean_ is the mean CT number in the ROI, and ROI_area_ is the total area (cm^2^) of the ROI and then averaged across all scan slices.

### 2.5 Calculation of SSDE

An SSDE derived from CT‐attenuation‐based calculations was calculated for each patient using the following equation, where a conversion factor calculated from *D*_w_ (Supporting Information) for a 32 cm diameter CTDI_vol_ reference phantom [8].

### 2.6 Analysis

To determine which of the four geometric size metrics (DV, LAT, the sum of DV and LAT, and the square root of the product of DV and LAT) best estimated the gold standard *D*_w_, the relationships between the effective diameters calculated using the four metrics and *D*_w_ were analyzed using linear regression.

SSDEs derived from CT‐attenuation‐based calculations and the relationship of SSDE with CTDI_vol_ were summarized separately for each patient body weight category (<10, ≥10–≤30, and >30 kg). To compare SSDE with published achievable doses (ADs) and diagnostic reference levels (DRLs) for SSDE, the median and 75th percentile of SSDE for all patients were calculated.

Body weight categories (<10, ≥10–≤30, and >30 kg) were examined for differences in SSDE with a nonparametric Kruskal–Wallis test. As the scan protocols were identical for all patients within each body weight category, the relationship between SSDE and body weight within each body weight category was analyzed with linear regression. Body weight was assessed for a linear relationship with SSDE using the inclusion of a squared term in the linear regression to test the linearity assumption.

All data analyses were completed under the supervision of an analytical epidemiologist using commercial software (Stata SE version 18, StataCorp, College Station, TX). A *p* value ≤0.05 was considered significant.

## 3 Results

### 3.1 Patient Population

Thirty dogs were included. The median age was 89 months (range, 3–180 months), and the median body weight for all animals was 20.8 kg (range, 2.6–60.7 kg). The median (range) of body weights across the three body weight categories was <10 kg, 6.9 kg (2.6–9.9 kg); ≥10–≤30 kg, 20.8 kg (12.6–28 kg); >30 kg, 44.4 kg (33–60.7 kg). There were 14 neutered females, 5 intact females, 9 neutered males, and 2 intact males. Breeds consisted of six mixed‐breed dogs, four Labrador Retrievers, three Siberian Huskies, two each of Cane Corsos and Yorkshire Terriers, and one each of Australian Shepherd, Bichon Frise, Boston Terrier, Chinese Crested, English Cocker Spaniel, French Bulldog, Great Dane, Pomeranian, Portuguese Water Dog, Pug, Rottweiler, Shiba Inu, and Standard Poodle. The median number of scans acquired per CT imaging episode was 5 (range, 1–10).

### 3.2 Geometric Size Metrics and *D*_w_

The median DV diameter for all dogs was 18.5 cm (range, 9.1–28.7 cm), and the median LAT diameter was 20.3 cm (range, 9.8–30.3 cm).

**FIGURE 3:**
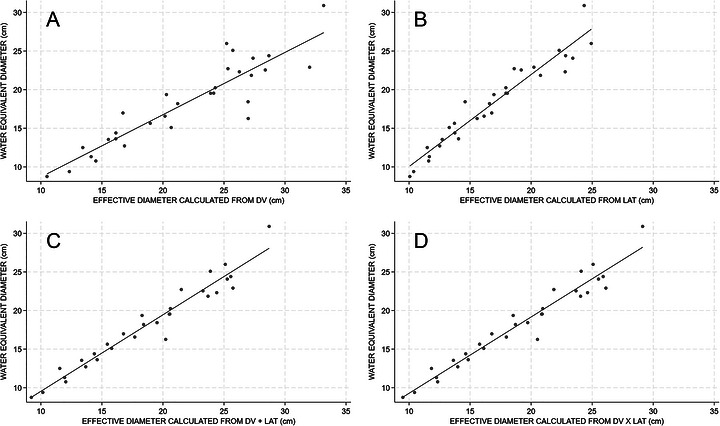
Scatter plots with linear fit lines showing the relationship between the effective diameters calculated using a single dimension measured from the CT scan (Graphs A and B) or a combination of both dimensions (Graphs C and D). Equations used to calculate effective diameter from the geometric metrics are shown in the Supporting Information. The adjusted *R*‐squared values for the effective diameters calculated on each metric were 0.81, dorsoventral (Graph A); 0.92, lateral (Graph B); 0.94, sum of dorsoventral and lateral (Graph C); and 0.94, product of dorsoventral and lateral (Graph D). Although the effective diameters calculated for all four metrics were significantly related to the water‐equivalent diameter (*D*_w_) (*p* < 0.001), the effective diameters calculated for the combinations of both dimensions better predicted *D*_w_. DV, dorsoventral; LAT, lateral.

The effective diameters based on all four metrics demonstrated statistically significant linear relationships with *D*_w_ (*p* < 0.001 for all models) (Table 1, Figure 3). The strongest correlation was observed for the combined DV and LAT dimensions (the sum of DV and LAT and the square root of the product of DV and LAT), with the highest adjusted *R* ^2^ values (0.94) and regression coefficients of 0.99 (95% CI: 0.93–1.09 and 0.93–1.08, respectively). The combined geometric size metrics demonstrated stronger predictive power than the single‐dimension metrics (DV or LAT).

**TABLE 1:** Linear regression analysis of geometric size metrics for estimating water‐equivalent diameter calculated from computed tomographic (CT) images.

Metric	Regression coefficient	95% CI	Intercept	95% CI	Adjusted *R*‐squared value	*p* value
DV diameter	0.80	0.65–0.95	0.72	−2.65 to 4.09	0.81	<0.001
LAT diameter	1.20	1.06–1.32	−1.89	−4.15 to 0.37	0.92	<0.001
DV + LAT diameter	0.99	0.90–1.09	−0.42	−2.23 to 1.39	0.94	<0.001
SQRT (DV × LAT)	0.99	0.90–1.08	−0.66	−2.51 to 1.19	0.94	<0.001

Abbreviations: CI, confidence interval; DV, dorsoventral diameter; LAT, lateral diameter; SQRT, square root.

The AAPM conversion factors to estimate SSDE using the DV and LAT dimensions measured using digital calipers in PACS are summarized in Table 2. Although both the sum of DV and LAT and the square root of their product showed equivalent predictive value for *D*_w_, conversion factors based on the sum are presented in Table 2 due to the simplicity of the calculation. The equations used to calculate the conversion factors are available in the Supporting Information.

**TABLE 2:** American Association of Physicists in Medicine conversion factors^a^ (CFs) as a function of the sum of the dorsoventral (DV) and lateral (LAT) diameters^b^ to estimate the size‐specific dose from the volume computed tomography dose index (CTDI_vol_) for canine abdominal computed tomographic (CT) scans, using the equation size‐specific dose estimate (SSDE) = CF × CTDI_vol_.

DV + LAT diameters (cm)	CF^+^	DV + LAT diameters (cm)	CF
15	2.84	38	1.87
16	2.79	39	1.83
17	2.74	40	1.80
18	2.69	41	1.77
19	2.64	42	1.74
20	2.59	43	1.71
21	2.55	44	1.67
22	2.50	45	1.64
23	2.46	46	1.62
24	2.41	47	1.59
25	2.37	48	1.56
26	2.32	49	1.53
27	2.28	50	1.50
28	2.24	51	1.47
29	2.20	52	1.45
30	2.16	53	1.42
31	2.12	54	1.40
32	2.08	55	1.37
33	2.05	56	1.35
34	2.01	57	1.32
35	1.97	58	1.30
36	1.94	59	1.27
37	1.90	60	1.25

*Note*: These conversion factors should only be used when the CTDIvol is based on a 32 cm diameter body dosimetry phantom.

^a^Equations used to calculate the conversion factors are available in Supporting Information.

^b^The DV and LAT dimensions are measured using digital calipers in PACS as the maximum diameter of the patient in the dorsal‐to‐ventral (DV) and right‐to‐left (LAT) directions, on the most cranial transverse slice on which the transverse process of the first lumbar vertebra was visible.

### 3.3 SSDE and Body Weight

SSDE for the three body weight categories and their relationship with CTDI_vol_ are summarized in Table 3. The median SSDE for all animals was 20 mGy (range, 16–23 mGy), and the 75th percentile of SSDE for all animals was 21 mGy.

**TABLE 3:** Size‐specific dose estimates in milligray (mGy) derived from CT‐attenuation‐based calculations ($\mathrm{SSD}E_{D_{w}}$) and their relationship with the volumetric computed tomographic dose index (CTDI_vol_) for 30 dogs undergoing computed tomography of the abdomen using weight‐based CT scan protocols.

	$\mathrm{SSD}E_{D_{w}}$ (mGy)			$\mathrm{SSD}E_{D_{w}}$/CTDI_vol_		
Weight category (kg)	Minimum	Median	Maximum	Minimum	Median	Maximum
<10	18	20	23	2.1	2.3	2.7
≥10–≤30	18	19	22	1.8	1.9	2.1
>30	16	21	23	1.2	1.6	1.7

**FIGURE 4:**
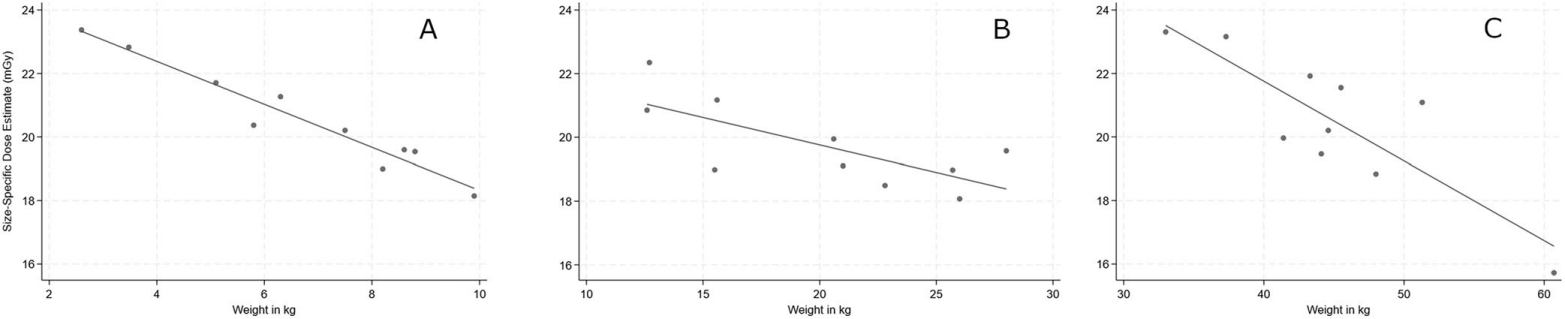
Scatter plots with linear fit lines showing the relationship between the size‐specific dose estimate (SSDE) and body weight, within each body weight category used to select the CT scan protocol. Note that the *x*‐axis scales differ between panels according to the body weight range represented in each category. The adjusted *R*‐squared values for the weight categories were 0.93, <10 kg (Graph A); 0.48, ≥10–≤30 kg (Graph B); and 0.68, >30 kg (Graph C). Although the selection of scan protocol by body weight resulted in no difference in SSDE between the body weight categories, body weight and dose were negatively correlated within each category (*p* values <0.001, 0.02, and 0.002 for the <10 kg (Graph A), ≥10–≤30 kg (Graph B), and >30 kg (Graph C) body weight categories, respectively).

There was no significant difference in SSDE between the three body size categories (*p* = 0.31). However, within each size category, there was a statistically significant negative correlation between body weight and estimated dose (*p* values <0.001, 0.02, and 0.002 for the <10, ≥10–≤30, and >30 kg body weight categories, respectively) (Table 4, Figure 4). Each of the tested relationships passed the linearity assumption test.

**TABLE 4:** Linear regression analysis of size‐specific dose estimates and body weight within weight‐based computed tomographic (CT) scan protocols.

Weight category (kg)	Coefficient	95% CI	Adjusted *R*‐squared value	*p* value
<10	−0.68	−0.82 to (−0.54)	0.93	<0.001
≥10–≤30	−0.17	−0.30 to (−0.04)	0.48	0.02
>30	−0.25	−0.38 to (−0.12)	0.68	0.002

Abbreviation: CI, confidence interval.

## 4 Discussion

This study found that the AAPM‐recommended approach for estimating SSDE can be applied to canine abdominal CT imaging and presents a practical and more reliable method for estimating individual patient radiation dose in a clinical veterinary setting. These findings pertain specifically to the abdominal region; for a given CTDI_vol_ and comparable anatomical dimensions, the thoracic region would receive a higher absorbed dose than the abdomen due to reduced x‐ray attenuation by the low‐density lung tissue. Future studies are needed to evaluate SSDE estimation in the canine thorax, where differences in tissue attenuation may affect the agreement between geometric size metrics and attenuation‐based dose estimates. This study did not evaluate human‐derived conversion factors for dogs; instead, it evaluated whether the AAPM SSDE approach, which uses geometric size metrics to approximate attenuation‐based dose estimates, can be applied to canine abdominal CT.

Conversion factors presented in Table 4 are specific to CTDI_vol_ measured using a 32 cm diameter phantom and the sum of DV and LAT dimensions. The displayed CTDI_vol_ is calculated using a standardized method across all CT scanner models and is based on manufacturer measurements from cylindrical polymethylmethacrylate phantoms [12]. A 32 cm diameter phantom is used for adult body examinations, and a 16 cm diameter phantom is used for adult and pediatric head examinations [12]. However, for reporting pediatric body examination CTDI_vol_, vendors vary in their use of phantom size, with some utilizing a 16 cm phantom and others a 32 cm phantom [12].

It has been found that combining the DV and LAT dimensions yields a more accurate estimate of the attenuation‐based metric *D*_w_ than either dimension alone, consistent with what has been reported for SSDE calculations for human pediatric abdominal CT scanning [9]. The same pediatric study also found CTDI_vol_ volumes based on a 32 cm phantom underestimated SSDE by approximately 50% for patients weighing 10–36 kg, which is comparable to the finding of this study. It was suggested that using a 16 cm phantom for all body CT exams in patients weighing less than 36 kg might eliminate the need for CTDI_vol_ to SSDE conversion factors. This is because CTDI_vol_ measured with a 16 cm phantom is approximately twice the value obtained from a 32 cm phantom, making it a closer match to SSDE for patients weighing less than 10 kg [9]. However, a conversion factor would still be required for patients weighing between 36 and 100 kg, regardless of which phantom was used [9].

The study cohort included dogs with varying thoracic conformations, including dolichomorphic and brachymorphic dogs, reflecting the diversity of breeds encountered in clinical practice. Body conformation was not categorized, as the primary objective was to evaluate geometric size metrics rather than breed‐ or shape‐specific subgroups. The combined use of DV and LAT dimensions would capture the variation in conformation, as differences in chest depth versus width are reflected in these measurements. This may explain the stronger agreement between combined geometric metrics and water‐equivalent diameter compared with single‐dimension metrics. The study did not have the power to evaluate whether specific body shapes resulted in better agreement between SSDE based on geometric metrics and *D*_w_‐based SSDE.

The AAPM 204 report suggested SSDE (in mGy) be reported by radiologists, along with the following supporting details: The diameter of the PMMA phantom used to generate the scanner‐displayed CTDI_vol_, the geometric size metric used to choose the conversion factor, the reference source for the conversion factor, and an estimate of the magnitude of the uncertainty associated with the method (for human abdominal scans, a few %) [8, 11]. The report recommends that SSDE values greater than 5 mGy be reported as whole numbers, whereas values below 5 mGy be reported with one decimal place [8]. If multiple CT scans are performed (e.g., pre‐ and post‐contrast scans), the dose estimate to the abdomen should be reported as the sum of the dose estimates for all the scans.

To illustrate how SSDEs can be incorporated into clinical practice, we provide a practical example using a canine abdominal CT examination. Following image acquisition, patient dimensions can be obtained using the digital calipers in a PACS system. In the patient shown in Figure 1, the maximum DV and right‐to‐left (LAT) dimensions were measured on the most cranial transverse slice on which the full transverse process of the first lumbar vertebra was visible. The DV dimension was 19.7 cm, and the LAT dimension was 22.0 cm. The sum of the two values is 41.7 cm. The CTDI_vol_ reported by the scanner for this examination (based on a 32 cm reference phantom) was 10.5 mGy. Using the size‐dependent conversion factors provided in AAPM Report 204 [8] (Table 2), a summed diameter of approximately 42 cm corresponds to a conversion factor of 1.74. The resulting SSDE for this abdominal CT examination is therefore 18.3 mGy (10.5 × 1.74 mGy). Uncertainties associated with this method are on the order of a few percentages. Reporting SSDE rather than CTDI_vol_ alone allows dose estimates to be compared across patients of different sizes and provides a clinically relevant method for protocol optimization and dose tracking in veterinary practice.

Veterinary CT examinations frequently include multiple acquisition phases and/or multiple body regions. The present study aimed to evaluate the applicability of geometric size‐based SSDE estimation for a single anatomic region, the abdomen, using a clinically practical approach. As recommended by AAPM Task Group reports 204 and 220, when multiple scan phases or body regions are acquired, SSDE should be calculated separately for each individual scan and the resulting dose estimates summed to estimate the total patient dose [8, 11]. Future studies to evaluate the use of geometric size metrics for SSDE estimation in thoracic and other regions are needed to estimate the full patient dose.

Although SSDE is a valuable tool for estimating patient‐specific dose in CT, it has several limitations that affect accuracy. SSDE does not consider organ dose distribution or scan length and therefore cannot be used to estimate effective dose (in mSv) or individual patient radiation risk [8]. A limitation of the SSDE estimation method described in this study is that it relied on image data from the completed CT scan, therefore providing dose information retrospectively. The AAPM has proposed estimating SSDE prospectively using geometric dimensions obtained from the CT localizer radiograph (scout image) to estimate SSDE; however, this approach was not evaluated in the current canine cohort [8]. The dose from a CT scan is highest in the center of the scan volume than at the ends of the volume; because CTDI_vol_ is an estimate of dose at the center of the scan volume (along the *z*‐axis), SSDE values derived from CTDI_vol_ will be slightly overestimated compared to the true dose to the entire volume [7].

Body condition score was not recorded and was not included as an independent variable in the dose estimation models. If two dogs had similar DV and LAT dimensions but differed in body condition (lean versus obese), geometric size metrics would provide similar SSDE values, whereas *D*_w_ would likely be different, as it also incorporates tissue attenuation and adipose tissue is less attenuating than soft tissue. However, AAPM Report 220 reported that the percentage difference between effective diameter (a geometric metric) and water‐equivalent diameter (which considers both geometry and attenuation) was only a few percent for abdomen phantoms [11]. This supports the use of geometric metrics for the estimation of abdominal CT dose across a range of body conditions.

Smaller patients receive higher radiation doses when identical CT scanner acquisition parameters are used [13, 14]. To mitigate this, our institution employs a body weight‐based protocol that adjusts the tube current–rotation time product (mAs) according to predefined weight categories to mitigate higher radiation doses in smaller patients. Using this approach, no significant differences in SSDE were observed among the three body weight categories (<10, ≥10–≤30, and >30 kg). As expected, a significant inverse relationship was observed between body weight and SSDE within each body weight category, as identical technical scan parameters were used.

The International Commission on Radiological Protection developed the DRL—the 75th percentile of the dose distribution in widespread use—to identify medical imaging procedures for which radiation dose levels are too high so that patient protection can be optimized [15]. A more stringent benchmark, the AD—the 50th percentile of the dose distribution—was proposed by the National Commission on Radiological Protection to further guide dose reduction [16]. In the United States, both DRL and AD for single‐phase pediatric abdominal CT scans have been established using data from the American College of Radiology Dose Index Registry [17]. For SSDE, the published DRLs for single‐phase, non‐contrast‐enhanced abdomen and pelvis CT scans within the size categories represented in the current study ranged from 5.4 to 14 mGy, whereas the corresponding ADs ranged from 4.6 to 11 mGy [17]. In comparison, the 50th and 75th percentiles of SSDE values in this canine cohort were approximately two to four times higher than these human benchmarks, suggesting a potential need for dose optimization in the abdominal CT protocols at this institution.

In conclusion, geometric size metrics measured from a single CT image were in close agreement with attenuation‐based SSDE (*D*_w_) for canine abdominal CT scans, supporting the feasibility of applying the AAPM SSDE estimation method to canine abdominal CT imaging. Scanner‐reported CTDI_vol_ underestimated patient dose by approximately 50%, demonstrating the importance of incorporating individual patient size into dose estimation. The study found that using a combination of DV and LAT dimensions, either by summing them or applying the square root of their product, provided more accurate SSDE estimates than either dimension alone. The SSDE values for canine patients undergoing abdominal CT scans at this institution were approximately two to four times higher than published human DRLs and ADs, indicating the potential need for optimization of patient dose.

## Author Contributions

Conception and design: Claire Whittaker, Monique Mayer, Matthew Hutcheson, and Sally Sukut. Acquisition of data: Claire Whittaker, Monique Mayer, and Sally Sukut. Analysis and interpretation of data: Claire Whittaker, Monique Mayer, Matthew Hutcheson, and Sally Sukut. Drafting the article: Claire Whittaker and Monique Mayer. Revising article for intellectual content: Claire Whittaker, Monique Mayer, Matthew Hutcheson, and Sally Sukut. Final approval of the completed article: Claire Whittaker, Monique Mayer, Matthew Hutcheson, and Sally Sukut.

## Disclosure

No reporting checklist was used.

## Conflicts of Interest

The authors declare no conflicts of interest.

## Supporting information




**Supporting File**: vru70223‐sup‐0001‐SuppMat.docx

## Data Availability

The data that support the findings of this study are available from the corresponding author upon reasonable request.
