# Supplementary material for: Patient Dimensions From a Single CT Image Provide a Clinically Practical and Accurate Method for Dose Estimation in Canine Abdominal CT Imaging
Source: Vet Radiol Ultrasound. 2026 Jul 29;67(5):e70223. doi: 10.1111/vru.70223 (PMC13416426; doi:10.1111/vru.70223)
Supplement: Supplementary file 1 — Supporting File: vru70223‐sup‐0001‐SuppMat.docx [file VRU-67-0-s001.docx]

To calculate the effective diameter from LAT, DV, and the sum of LAT and DV, the following equation was used:

$$y=a+bx+cx^{2}$$

Where the values for a, b and c are:

| **x parameter** | **y parameter** | **a** | **b** | **c** |
| --- | --- | --- | --- | --- |
| LAT dimension (cm) | Effective diameter | 5.899298E0 | 3.270494E-1 | 9.978896E-3 |
| DV dimension (cm) | Effective diameter | -3.744858E0 | 1.671734E0 | -1.338955E-2 |
| LAT + DV (cm) | Effective diameter | -2.03128E-1 | 4.958912E-1 | 0 |

To calculate the conversion factor (y parameter) from the effective diameter (x parameter), the following equation was used, where a = 3.704369, and b = 0.03671937:

$$y=a \times e^{-bx}$$
